# Supplementary material for: Apoptosis of Pancreatic Cancer Cells after Co-Treatment with Eugenol and Tumor Necrosis Factor-Related Apoptosis-Inducing Ligand
Source: Cancers (Basel). 2024 Sep 5;16(17):3092. doi: 10.3390/cancers16173092 (PMC11394607; doi:10.3390/cancers16173092)
Supplement: Supplementary file 1 [file cancers-16-03092-s001.zip › cancers-3126313-supplementary.pdf]

Supplementary Figure S1. The original Western blot figures.

For all Western blot figures,
densitometry readings/intensity ratio of each band.

Fig. 3d

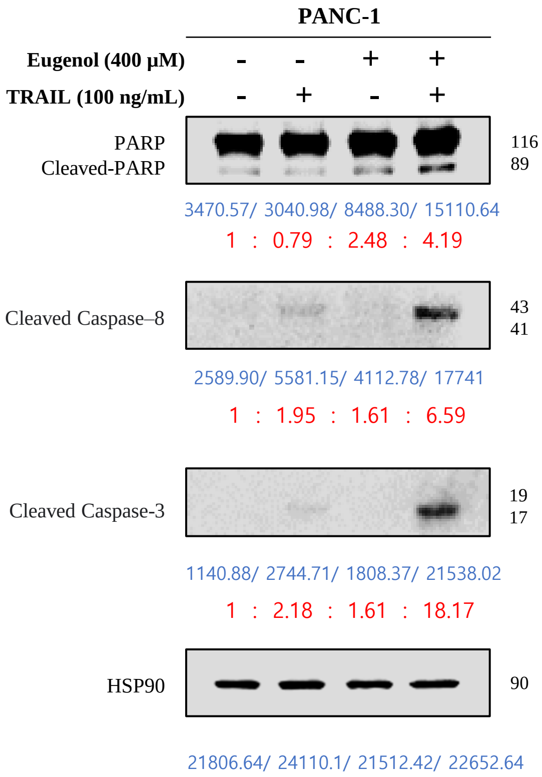

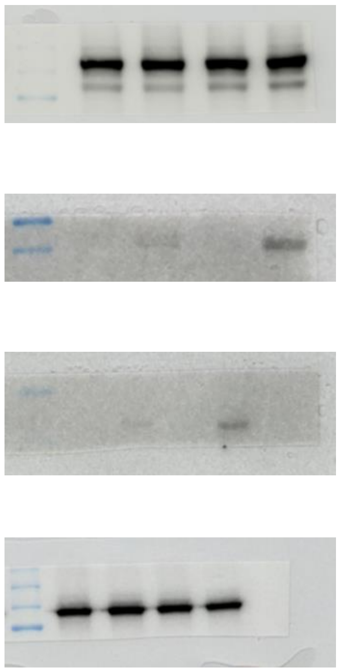

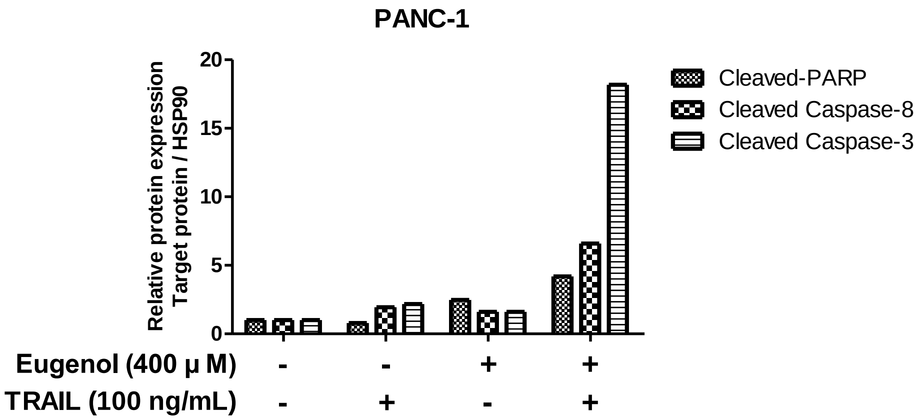

Fig. 4a

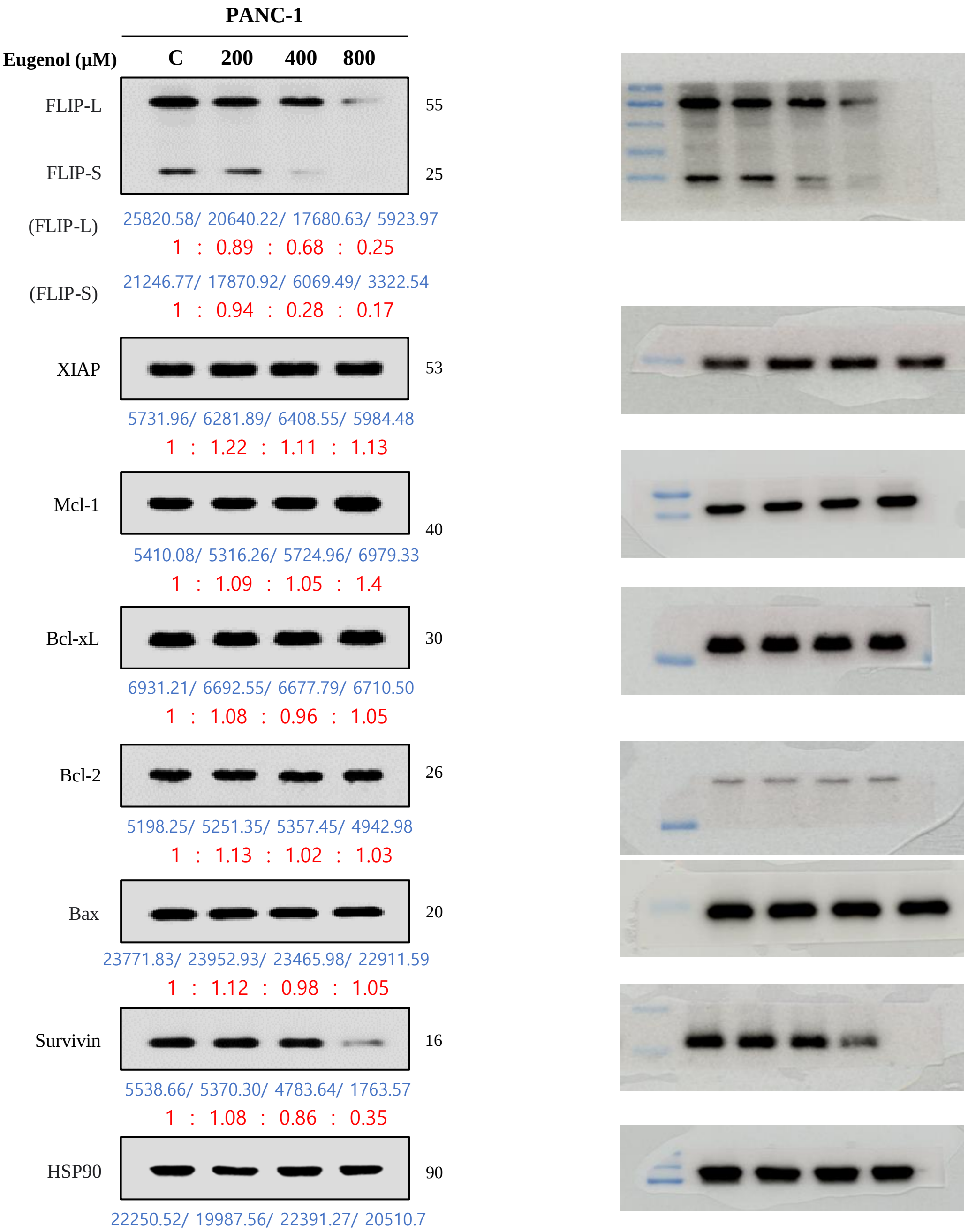

PANC-1
Eugenol (μM)
C
200
400
800
FLIP-L
55
FLIP-S
25
(FLIP-L)
25820.58/ 20640.22/ 17680.63/ 5923.97
1 : 0.89 : 0.68 : 0.25
(FLIP-S)
21246.77/ 17870.92/ 6069.49/ 3322.54
1 : 0.94 : 0.28 : 0.17
XIAP
53
5731.96/ 6281.89/ 6408.55/ 5984.48
1 : 1.22 : 1.11 : 1.13
Mcl-1
40
5410.08/ 5316.26/ 5724.96/ 6979.33
1 : 1.09 : 1.05 : 1.4
Bcl-xL
30
6931.21/ 6692.55/ 6677.79/ 6710.50
1 : 1.08 : 0.96 : 1.05
Bcl-2
26
5198.25/ 5251.35/ 5357.45/ 4942.98
1 : 1.13 : 1.02 : 1.03
Bax
20
23771.83/ 23952.93/ 23465.98/ 22911.59
1 : 1.12 : 0.98 : 1.05
Survivin
16
5538.66/ 5370.30/ 4783.64/ 1763.57
1 : 1.08 : 0.86 : 0.35
HSP90
90
22250.52/ 19987.56/ 22391.27/ 20510.7

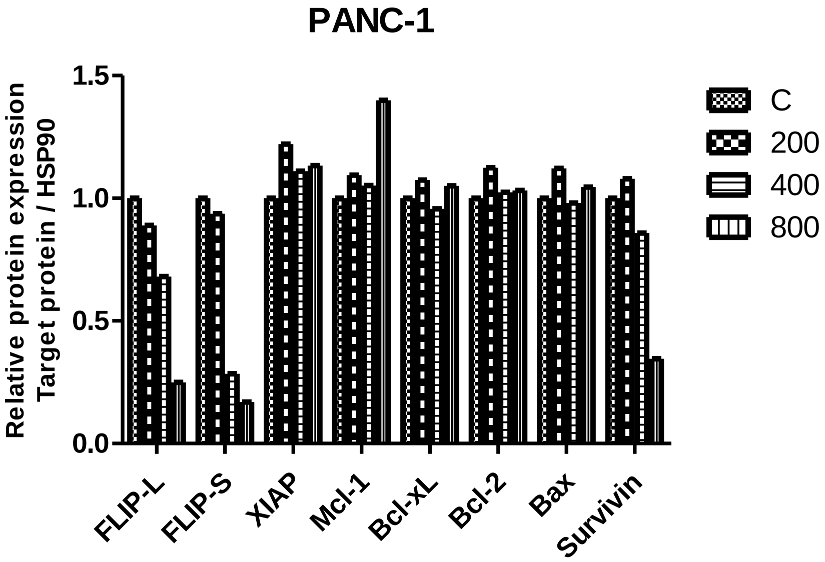

PANC-1
Relative protein expression
Target protein / HSP90
1.5
1.0
0.5
0.0
FLIP-L
FLIP-S
XIAP
Mcl-1
Bcl-xL
Bcl-2
Bax
Survivin
C
200
400
800

Fig. 4b

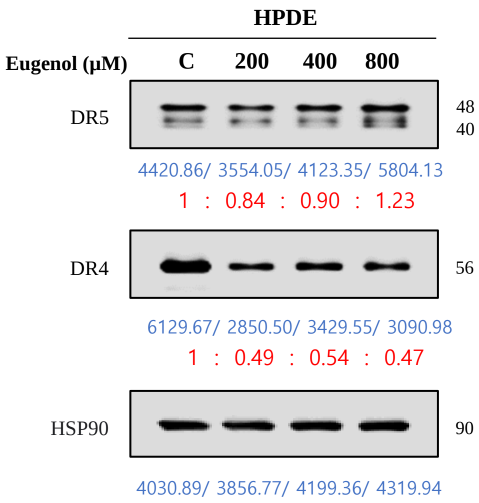

HPDE
Eugenol (μM)
C
200
400
800
DR5
48
40
4420.86/ 3554.05/ 4123.35/ 5804.13
1 : 0.84 : 0.90 : 1.23
DR4
56
6129.67/ 2850.50/ 3429.55/ 3090.98
1 : 0.49 : 0.54 : 0.47
HSP90
90
4030.89/ 3856.77/ 4199.36/ 4319.94

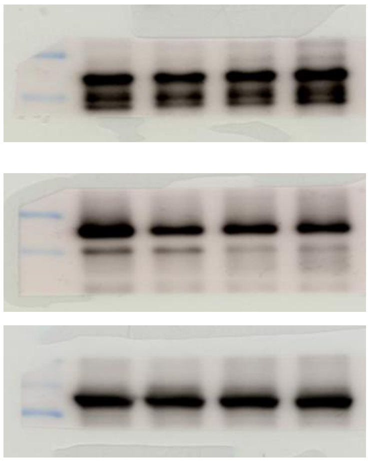

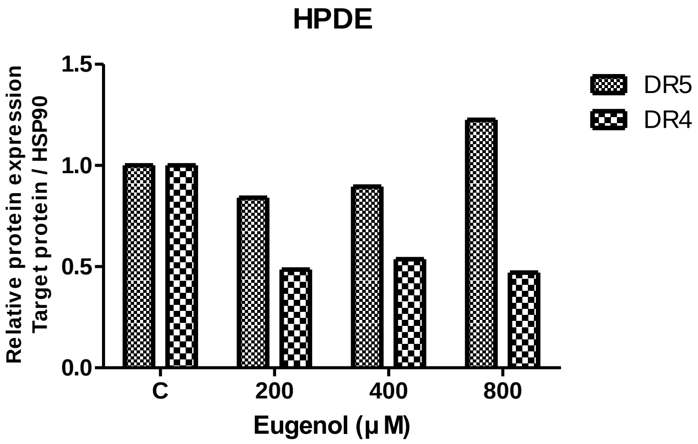

HPDE
Relative protein expression
Target protein / HSP90
1.5
1.0
0.5
0.0
C
200
400
800
Eugenol (μ M)
DR5
DR4

Fig. 4c

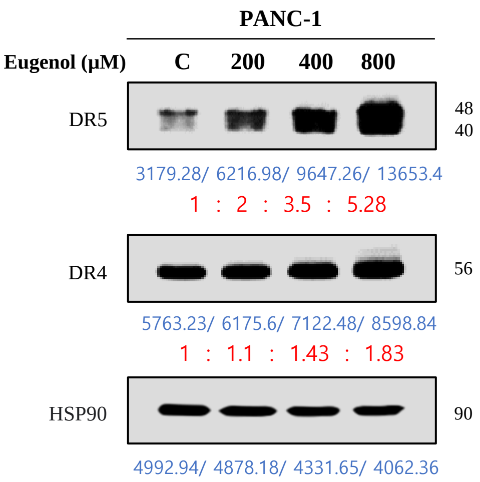
PANC-1
Eugenol (μM) C 200 400 800
DR5
48
40
3179.28/ 6216.98/ 9647.26/ 13653.4
1 : 2 : 3.5 : 5.28
DR4
56
5763.23/ 6175.6/ 7122.48/ 8598.84
1 : 1.1 : 1.43 : 1.83
HSP90
90
4992.94/ 4878.18/ 4331.65/ 4062.36

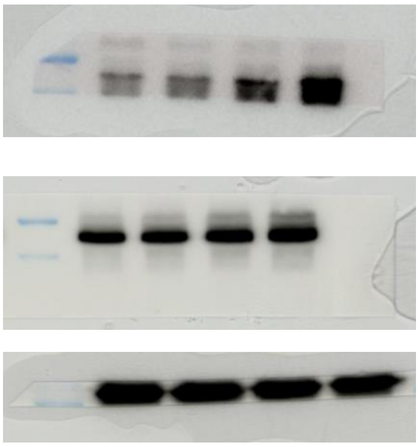

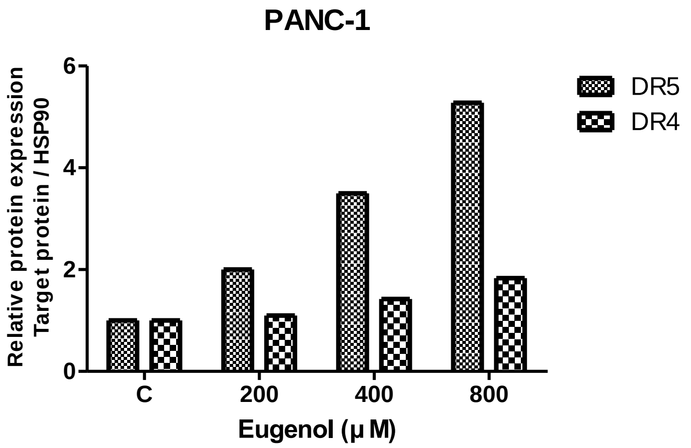
PANC-1
Relative protein expression
Target protein / HSP90
0
2
4
6
C
200
400
800
Eugenol (μ M)
DR5
DR4

Fig. 4f

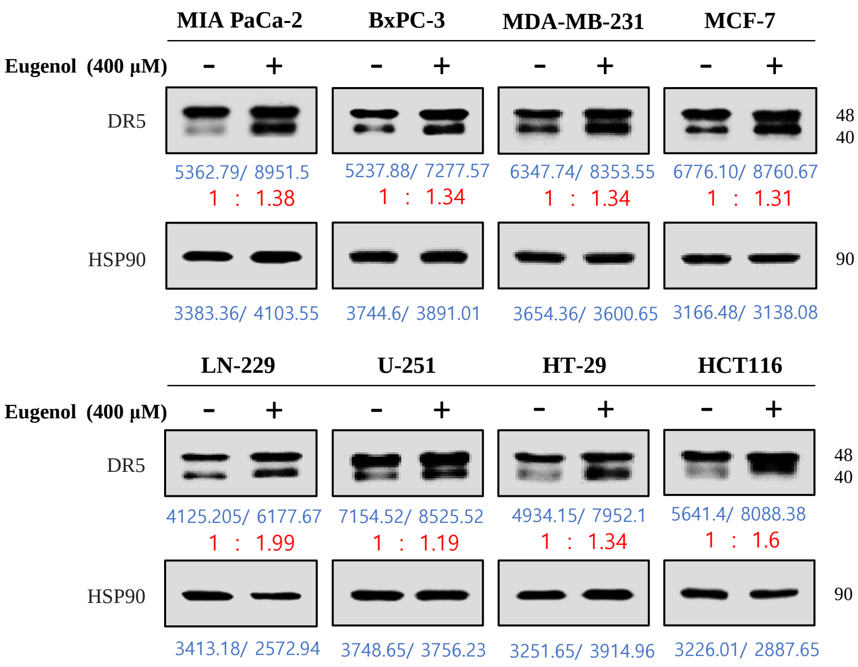
MIA PaCa-2
BxPC-3
MDA-MB-231
MCF-7
Eugenol (400 µM)
- + - + - + - +
DR5
48
40
5362.79/ 8951.5
1 : 1.38
5237.88/ 7277.57
1 : 1.34
6347.74/ 8353.55
1 : 1.34
6776.10/ 8760.67
1 : 1.31
HSP90
90
3383.36/ 4103.55
3744.6/ 3891.01
3654.36/ 3600.65
3166.48/ 3138.08
LN-229
U-251
HT-29
HCT116
Eugenol (400 µM)
- + - + - + - +
DR5
48
40
4125.205/ 6177.67
1 : 1.99
7154.52/ 8525.52
1 : 1.19
4934.15/ 7952.1
1 : 1.34
5641.4/ 8088.38
1 : 1.6
HSP90
90
3413.18/ 2572.94
3748.65/ 3756.23
3251.65/ 3914.96
3226.01/ 2887.65

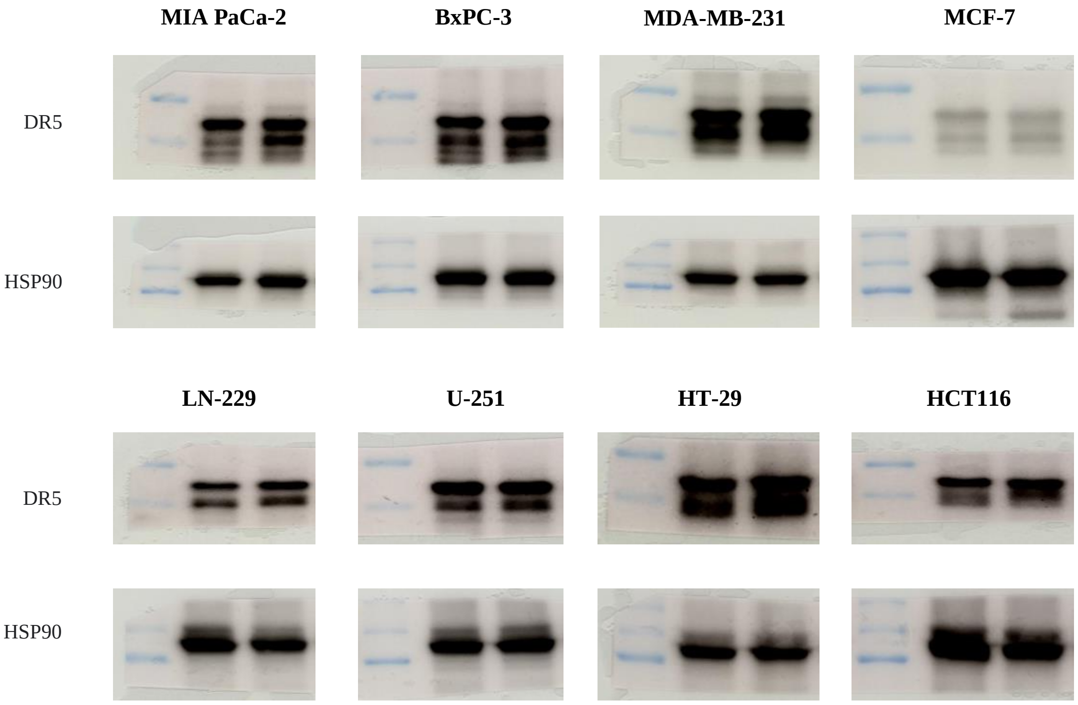
MIA PaCa-2
BxPC-3
MDA-MB-231
MCF-7
DR5
HSP90
LN-229
U-251
HT-29
HCT116
DR5
HSP90

Fig. 5a

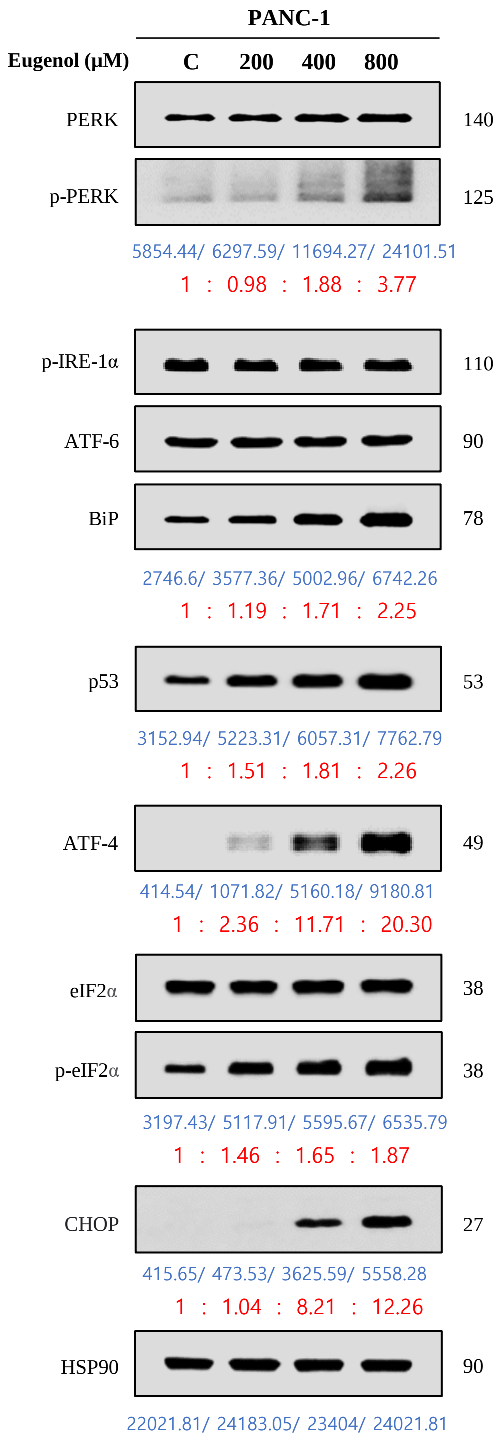
PANC-1
Eugenol (μM) C 200 400 800
PERK 140
p-PERK 125
5854.44/ 6297.59/ 11694.27/ 24101.51
1 : 0.98 : 1.88 : 3.77
p-IRE-1α 110
ATF-6 90
BiP 78
2746.6/ 3577.36/ 5002.96/ 6742.26
1 : 1.19 : 1.71 : 2.25
p53 53
3152.94/ 5223.31/ 6057.31/ 7762.79
1 : 1.51 : 1.81 : 2.26
ATF-4 49
414.54/ 1071.82/ 5160.18/ 9180.81
1 : 2.36 : 11.71 : 20.30
eIF2α 38
p-eIF2α 38
3197.43/ 5117.91/ 5595.67/ 6535.79
1 : 1.46 : 1.65 : 1.87
CHOP 27
415.65/ 473.53/ 3625.59/ 5558.28
1 : 1.04 : 8.21 : 12.26
HSP90 90
22021.81/ 24183.05/ 23404/ 24021.81

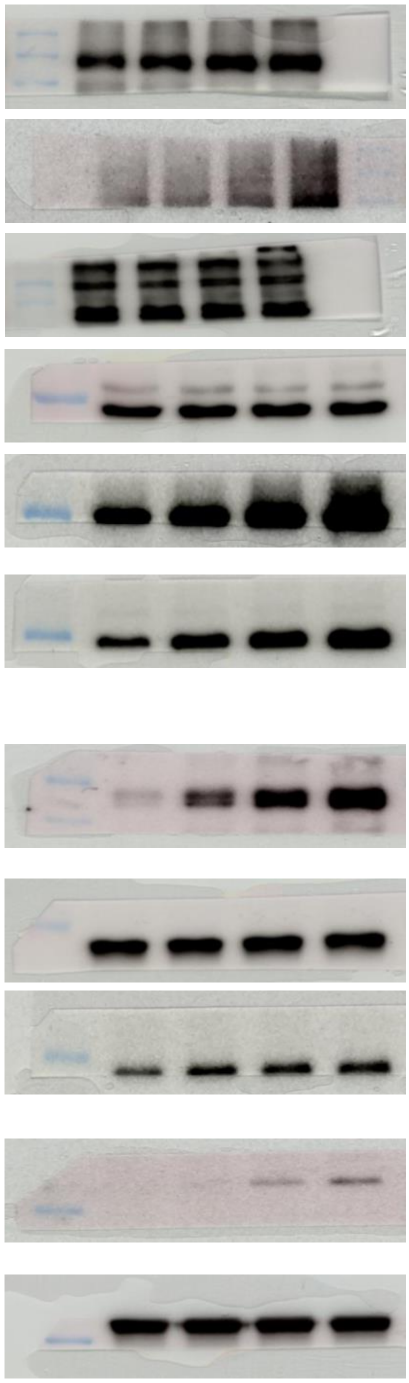

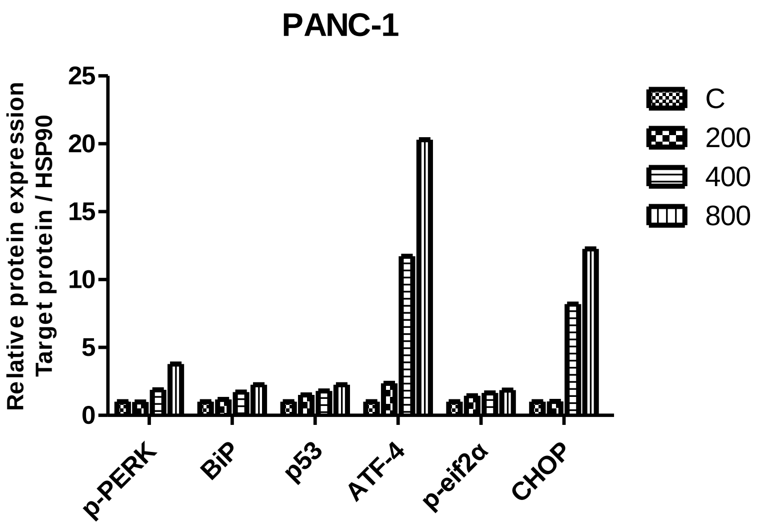
PANC-1
Relative protein expression
Target protein / HSP90
0
5
10
15
20
25
p-PERK
BiP
p53
ATF-4
p-eif2α
CHOP
C
200
400
800

Fig. 6c

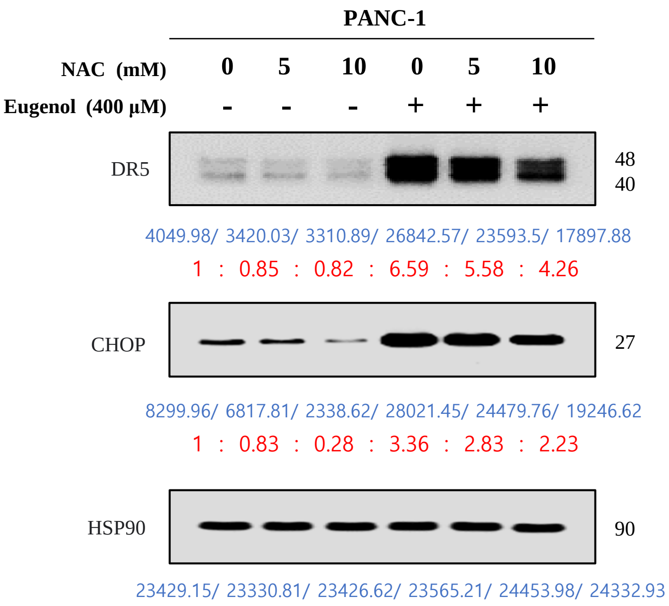
PANC-1
NAC (mM) 0 5 10 0 5 10
Eugenol (400 µM) - - - + + +
DR5
48
40
4049.98/ 3420.03/ 3310.89/ 26842.57/ 23593.5/ 17897.88
1 : 0.85 : 0.82 : 6.59 : 5.58 : 4.26
CHOP
27
8299.96/ 6817.81/ 2338.62/ 28021.45/ 24479.76/ 19246.62
1 : 0.83 : 0.28 : 3.36 : 2.83 : 2.23
HSP90
90
23429.15/ 23330.81/ 23426.62/ 23565.21/ 24453.98/ 24332.93

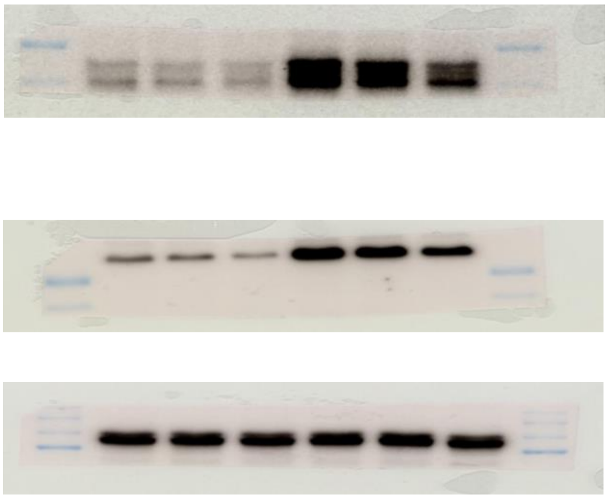

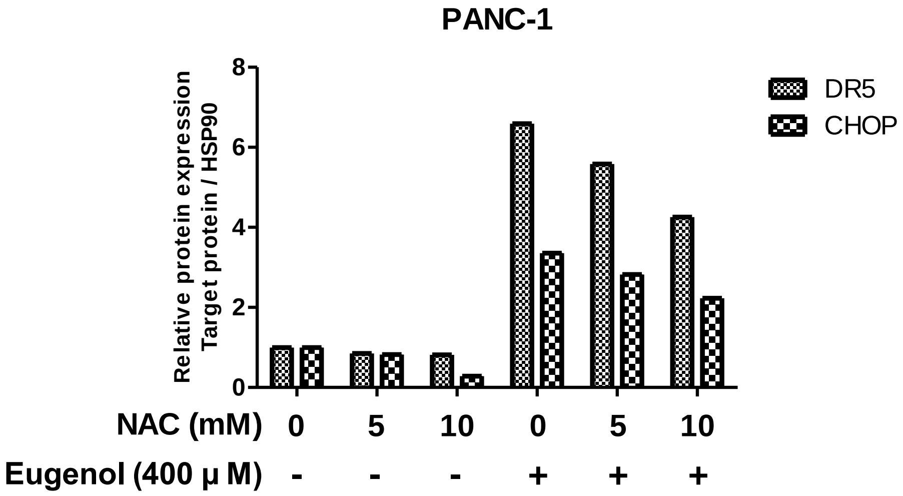
PANC-1
Relative protein expression
Target protein / HSP90
0
2
4
6
8
DR5
CHOP
NAC (mM) 0 5 10 0 5 10
Eugenol (400 μ M) - - - + + +

Fig. 6d

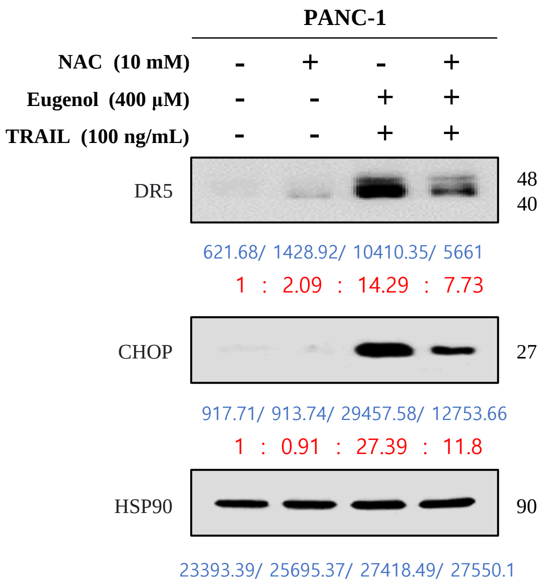
PANC-1
NAC (10 mM) - + - +
Eugenol (400 μM) - - + +
TRAIL (100 ng/mL) - - + +
DR5
48
40
621.68/ 1428.92/ 10410.35/ 5661
1 : 2.09 : 14.29 : 7.73
CHOP
27
917.71/ 913.74/ 29457.58/ 12753.66
1 : 0.91 : 27.39 : 11.8
HSP90
90
23393.39/ 25695.37/ 27418.49/ 27550.1

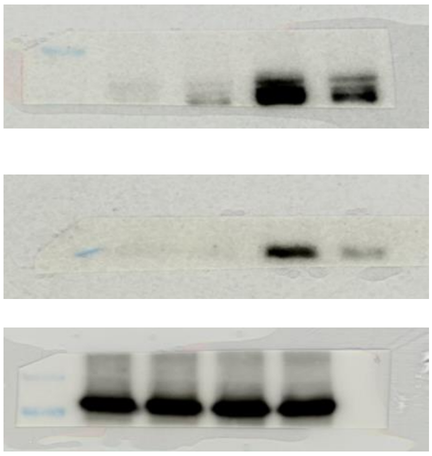

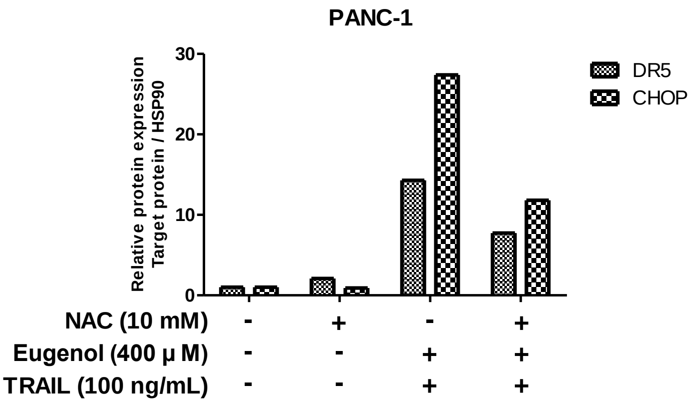
PANC-1
Relative protein expression
Target protein / HSP90
30
20
10
0
DR5
CHOP
NAC (10 mM) - + - +
Eugenol (400 μ M) - - + +
TRAIL (100 ng/mL) - - + +

Fig. 6e

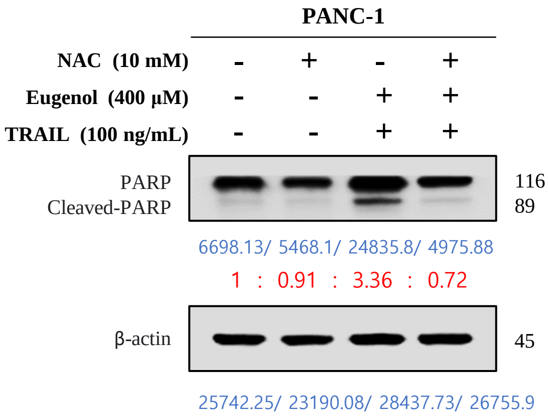
PANC-1
NAC (10 mM) - + - +
Eugenol (400 μM) - - + +
TRAIL (100 ng/mL) - - + +
PARP
Cleaved-PARP
116
89
6698.13/ 5468.1/ 24835.8/ 4975.88
1 : 0.91 : 3.36 : 0.72
β-actin
45
25742.25/ 23190.08/ 28437.73/ 26755.9

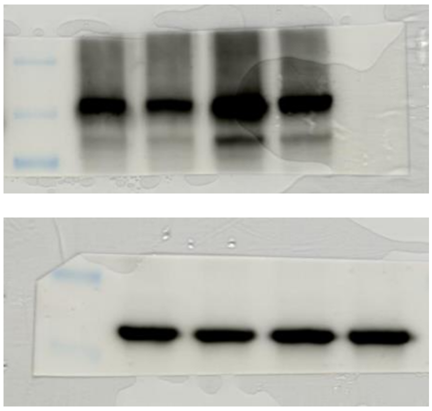

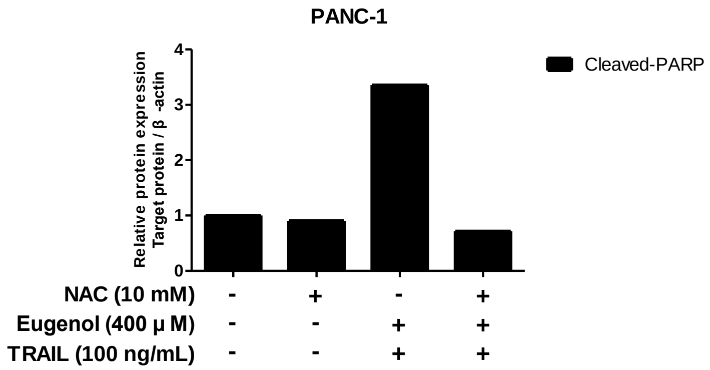
PANC-1
Relative protein expression
Target protein / β -actin
4
3
2
1
0
Cleaved-PARP
NAC (10 mM) - + - +
Eugenol (400 μ M) - - + +
TRAIL (100 ng/mL) - - + +

Fig. 7a

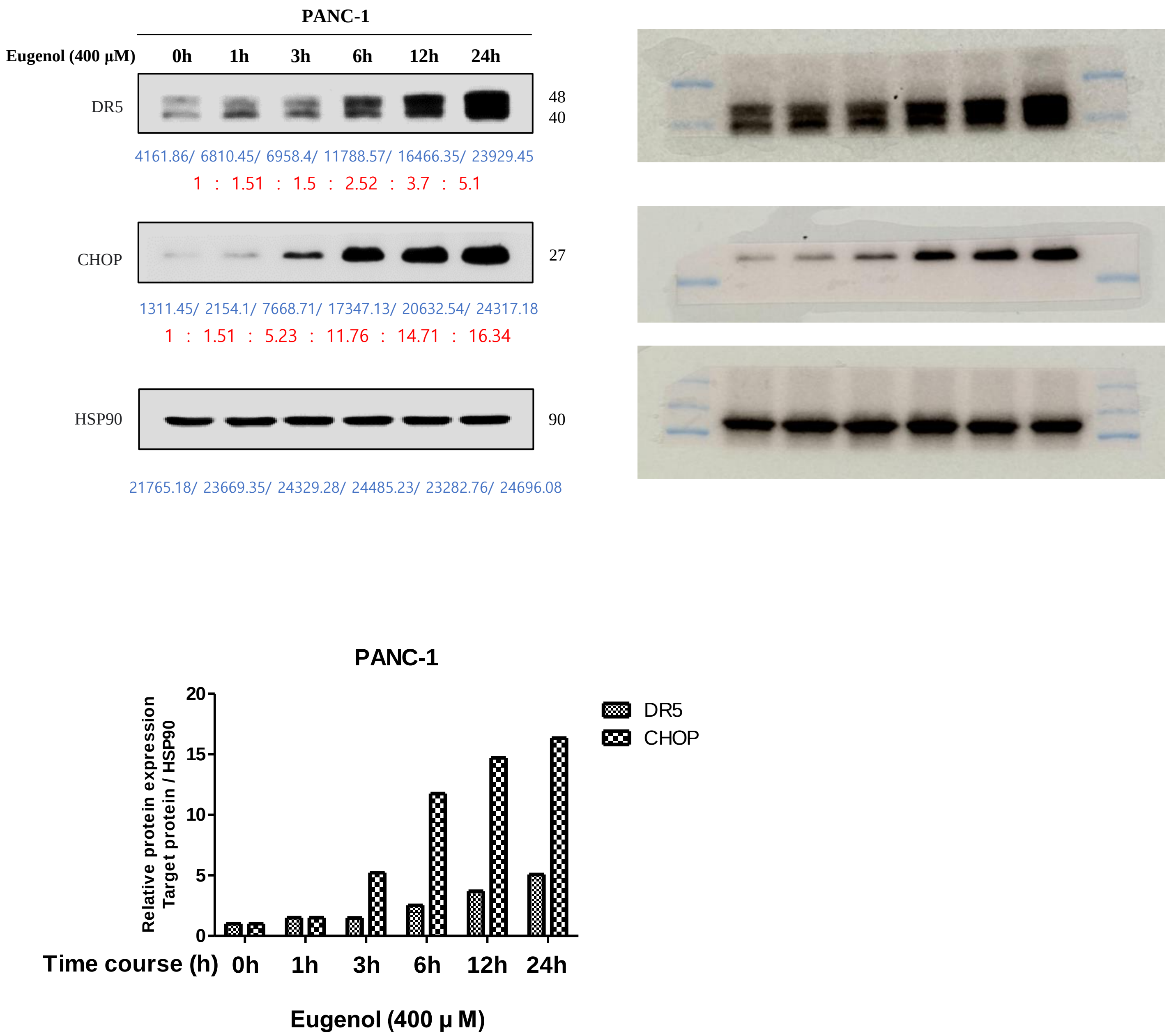
PANC-1
Eugenol (400 μM)
0h
1h
3h
6h
12h
24h
DR5
48
40
4161.86/ 6810.45/ 6958.4/ 11788.57/ 16466.35/ 23929.45
1 : 1.51 : 1.5 : 2.52 : 3.7 : 5.1
CHOP
27
1311.45/ 2154.1/ 7668.71/ 17347.13/ 20632.54/ 24317.18
1 : 1.51 : 5.23 : 11.76 : 14.71 : 16.34
HSP90
90
21765.18/ 23669.35/ 24329.28/ 24485.23/ 23282.76/ 24696.08
PANC-1
Relative protein expression
Target protein / HSP90
20
15
10
5
0
DR5
CHOP
Time course (h)
0h
1h
3h
6h
12h
24h
Eugenol (400 μ M)

Fig. 7b

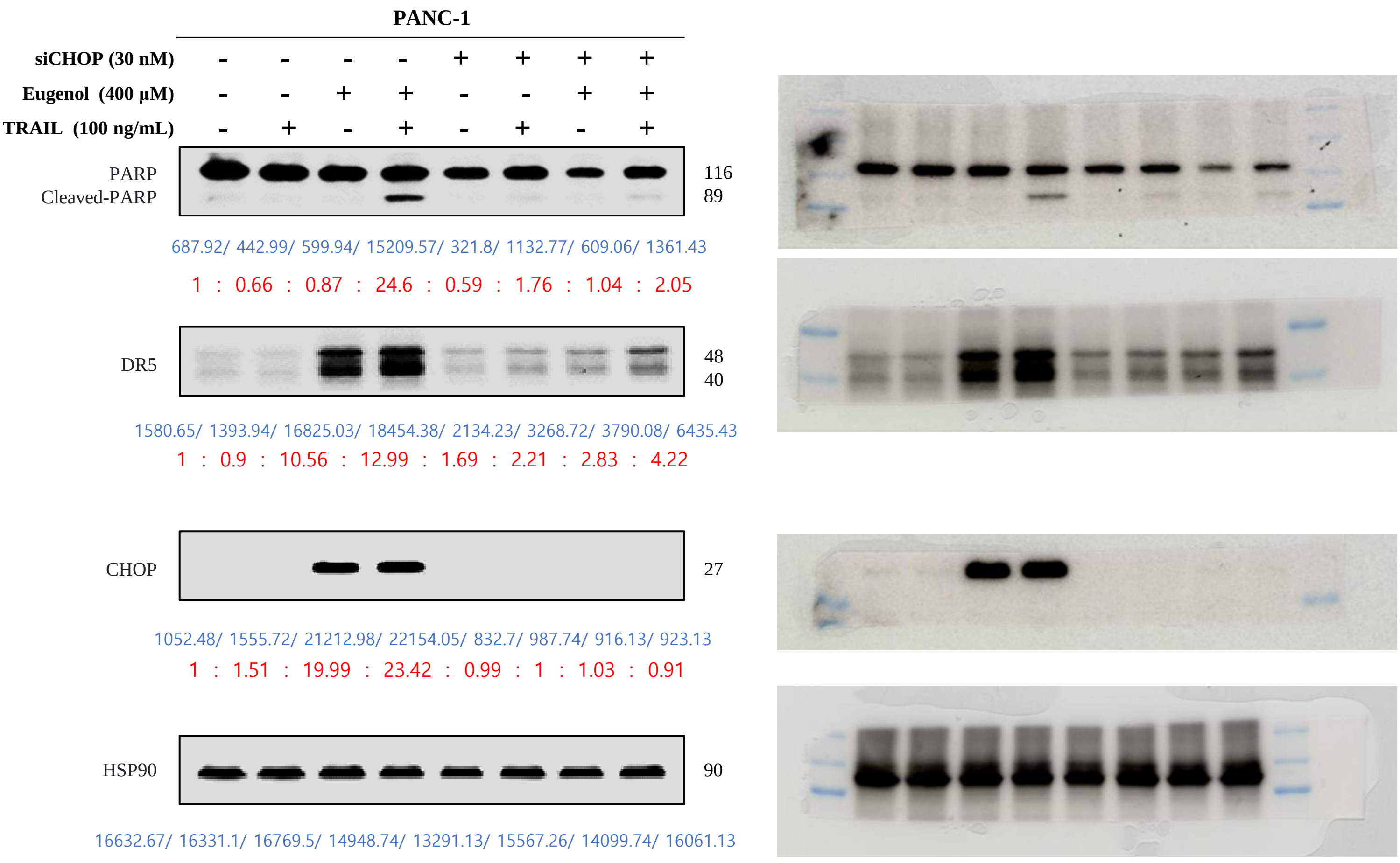
PANC-1
siCHOP (30 nM) - - - - + + + +
Eugenol (400 µM) - - + + - - + +
TRAIL (100 ng/mL) - + - + - + - +
PARP
Cleaved-PARP
116
89
687.92/ 442.99/ 599.94/ 15209.57/ 321.8/ 1132.77/ 609.06/ 1361.43
1 : 0.66 : 0.87 : 24.6 : 0.59 : 1.76 : 1.04 : 2.05
DR5
48
40
1580.65/ 1393.94/ 16825.03/ 18454.38/ 2134.23/ 3268.72/ 3790.08/ 6435.43
1 : 0.9 : 10.56 : 12.99 : 1.69 : 2.21 : 2.83 : 4.22
CHOP
27
1052.48/ 1555.72/ 21212.98/ 22154.05/ 832.7/ 987.74/ 916.13/ 923.13
1 : 1.51 : 19.99 : 23.42 : 0.99 : 1 : 1.03 : 0.91
HSP90
90
16632.67/ 16331.1/ 16769.5/ 14948.74/ 13291.13/ 15567.26/ 14099.74/ 16061.13

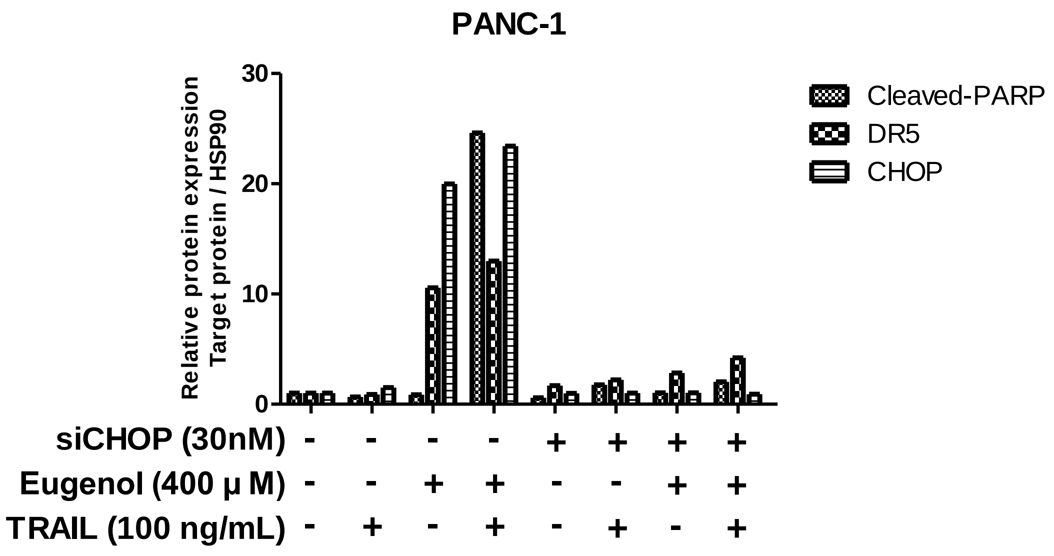
PANC-1
Relative protein expression
Target protein / HSP90
30
20
10
0
Cleaved-PARP
DR5
CHOP
siCHOP (30nM) - - - - + + + +
Eugenol (400 µ M) - - + + - - + +
TRAIL (100 ng/mL) - + - + - + - +
